# Diabetes type 2 risk gene Dusp8 is associated with altered sucrose reward behavior in mice and humans

**DOI:** 10.1002/brb3.1928

**Published:** 2020-11-01

**Authors:** Peter Baumann, Sonja C. Schriever, Stephanie Kullmann, Annemarie Zimprich, Andreas Peter, Valerie Gailus‐Durner, Helmut Fuchs, Martin Hrabe de Angelis, Wolfgang Wurst, Matthias H. Tschöp, Martin Heni, Sabine M. Hölter, Paul T. Pfluger

**Affiliations:** ^1^ Research Unit Neurobiology of Diabetes Helmholtz Zentrum München Neuherberg Germany; ^2^ Institute for Diabetes and Obesity Helmholtz Zentrum München Neuherberg Germany; ^3^ German Center for Diabetes Research (DZD) Neuherberg Germany; ^4^ Neurobiology of Diabetes TUM School of Medicine Technical University of Munich Munich Germany; ^5^ Institute for Diabetes Research and Metabolic Diseases (IDM) of the Helmholtz Center Munich at the University of Tübingen Tübingen Germany; ^6^ Department of Internal Medicine IV University Hospital of Tübingen Tübingen Germany; ^7^ German Mouse Clinic Institute of Experimental Genetics Helmholtz Zentrum München Neuherberg Germany; ^8^ Institute of Developmental Genetics Helmholtz Zentrum München Neuherberg Germany; ^9^ Chair of Developmental Genetics c/o Helmholtz Zentrum München Technische Universität München‐Weihenstephan Neuherberg Germany; ^10^ Institute for Clinical Chemistry and Pathobiochemistry Department for Diagnostic Laboratory Medicine University Hospital of Tübingen Tübingen Germany; ^11^ Chair of Experimental Genetics School of Life Science Weihenstephan Technische Universität München Freising Germany; ^12^ German Center for Neurodegenerative Diseases (DZNE) Site Munich Munich Germany; ^13^ Munich Cluster for Systems Neurology (SyNergy) Ludwig‐Maximilians‐Universität München Munich Germany; ^14^ Division of Metabolic Diseases Technische Universität München Munich Germany

**Keywords:** dopamine, Dusp8, MAP kinase, sucrose reward

## Abstract

**Background:**

Dusp8 is the first GWAS‐identified gene that is predominantly expressed in the brain and has previously been linked with the development of diabetes type 2 in humans. In this study, we unravel how Dusp8 is involved in the regulation of sucrose reward behavior.

**Methods:**

Female, chow‐fed global Dusp8 WT and KO mice were tested in an observer‐independent IntelliCage setup for self‐administrative sucrose consumption and preference followed by a progressive ratio task with restricted sucrose access to monitor seeking and motivation behavior. Sixty‐three human carriers of the major C and minor T allele of *DUSP8* SNP rs2334499 were tested for their perception of food cues by collecting a rating score for sweet versus savory high caloric food.

**Results:**

Dusp8 KO mice showed a comparable preference for sucrose, but consumed more sucrose compared to WT mice. In a progressive ratio task, Dusp8 KO females switched to a “trial and error” strategy to find sucrose while control Dusp8 WT mice kept their previously established seeking pattern. Nonetheless, the overall motivation to consume sucrose, and the levels of dopaminergic neurons in the brain areas NAcc and VTA were comparable between genotypes. Diabetes‐risk allele carriers of *DUSP8* SNP rs2334499 preferred sweet high caloric food compared to the major allele carriers, rating scores for savory food remained comparable between groups.

**Conclusion:**

Our data suggest a novel role for Dusp8 in the perception of sweet high caloric food as well as in the control of sucrose consumption and foraging in mice and humans.

## Graphical abstract

Sucrose reward studies in female mice deficient for the MAPK phosphatase dual specificity phosphatase 8 (Dusp8) revealed increased sucrose consumption, unaltered sucrose preference, and perturbed sucrose‐seeking behavior. Human carriers of DUSP8 diabetes‐risk SNP rs2334499 preferred sweet high caloric food compared to the major allele carriers. These data suggest a new role for Dusp8 in the perception of sweet high caloric food as well as in sucrose consumption and foraging.

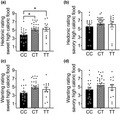

## 1 INTRODUCTION

The ever increasing prevalence of type 2 diabetes (T2D) has led to a surge in studies that explored the genetic underpinnings for this systemic disease. T2D‐associated loci identified in these genetic studies were mostly pointing toward peripheral pathologies such as an aberrant insulin secretion (Krentz & Gloyn, 2020). Few, if any loci hinted toward CNS‐based mechanisms, which remains surprising given the purported role of the brain as glucoregulatory organ (Kullmann et al., 2020; Ruud et al., 2017; Stemmer et al., 2019).

Recently, we revealed hypothalamic insulin resistance in human carriers of the minor T/T allele of SNP rs2334499 (Schriever et al., 2020), a frequent polymorphism previously associated with a modestly increased T2D risk (Kong et al., 2009; Morris et al., 2012). Based on the close proximity of SNP rs2334499 to the gene dual specificity phosphatase 8 (DUSP8), a likely relationship had been implicated (Kong et al., 2009; Morris et al., 2012). Consistent with that still unexplored assumption, we found hypothalamic insulin resistance and systemic glucose intolerance in high‐fat diet‐fed Dusp8 KO mice (Schriever et al., 2020). Mechanistically, we revealed an impaired HPA axis feedback control with chronic hypercorticosteronemia, and an altered sympathetic nervous system tone as driving factors for the dysfunctional glucose control in Dusp8 KO mice.

Dusp8 is a phosphatase specific for mitogen‐activated kinases (MAPK) that is predominantly expressed in the brain and to lesser extent in muscles and bone (Martell et al., 1995; Schriever et al., 2020). The specificity of Dusp8 toward MAPK appears to be tissue and context dependent. Dusp8 overexpressed in cells showed highest dephosphorylation activities toward Jnk and minor activities toward p38, and an absence of activity toward Erk (Muda et al., 1996; Schriever et al., 2020). In vivo, global Dusp8 deficient mice had Jnk hyperactivation in the hypothalamus (Schriever et al., 2020), but Erk hyperactivation in the hippocampus (Baumann et al., 2019) and the heart (Liu et al., 2016). These data are consistent with a complex crosstalk of MAPK that is finely regulated by numerous scaffold proteins and Dusp family members (Farooq & Zhou, 2004); they further suggest a prominent and tissue‐specific role for Dusp8 in MAPK signaling and function.

Recently, we reported decreased hippocampal volumes in human carriers of *DUSP8* minor allele rs2334499 (Baumann et al., 2019). The hippocampus is a mesolimbic brain area that is fundamentally involved in cognitive behaviors. Consistent with the human data, we found decreased hippocampus mass and perturbed anxiety, locomotion, and spatial cognition behaviors in mice with global Dusp8 deficiency (Baumann et al., 2019). The hippocampus is moreover a central brain area involved in food reward behaviors (Kanoski & Grill, 2017; Tracy et al., 2001) that is highly interconnected with dopaminergic centers within the basal ganglia via bidirectional as well as unidirectional glutamatergic and GABAergic projections (Thierry et al., 2000). Perturbations in this network, which further includes areas such as the prefrontal cortex or thalamus (Berthoud et al., 2011; Sescousse et al., 2013), have been linked to alterations in reward sensation and foraging behavior and an impulsive over‐consumption and binge‐eating of incentive sweet treats (Berridge et al., 2010; Castro & Berridge, 2014; Robinson & Berridge, 1993; Turton et al., 2017). Food reward nuclei of the limbic system are moreover in direct connection with homeostatic feeding circuits originating in the hypothalamus (Berthoud et al., 2011).

Here, driven by our recent work on hippocampal as well as hypothalamic Dusp8 function, we aimed to assess whether Dusp8 also plays a role in regulating feeding reward behaviors. Reward studies in rodents mostly entail the use of sucrose as incentive salience stimulus. These studies revealed a major role for dopamine in controlling sucrose reward behavior, but showed that these effects are largely confined to wanting behavior with limited effects on liking or conditioned learning (Berridge, 2007). Accordingly, we aimed to assess sucrose reward and wanting behaviors in Dusp8 KO mice using sucrose self‐administration tests, and interrogate whether Dusp8 deficiency perturbs dopaminergic reward circuits.

We further aimed to investigate the link between the human *DUSP8* diabetes‐risk variant and the preference for sweet high caloric versus savory high caloric foods. Sugar‐sweetened beverage (SSB) intake is a known risk factor for the development of diabetes, even independently from obesity (Fagherazzi et al., 2013; Imamura et al., 2016; Qi et al., 2012; Romaguera et al., 2013). Accordingly, we aimed to gain first insights on the presently unresolved question whether a genetic predisposition can affect food reward behaviors to exacerbate metabolic dysfunctions based on elevated sugar consumption.

## 2 METHODS

### 2.1 Animals

Dusp8 global WT and KO mice were generated as described (Liu et al., 2016). All mice in our studies were female littermates derived from heterozygous parents on a C57BL/6J background. Mice had free access to chow diet (Altromin, #1314) and were group‐housed on a 12:12 hr light–dark cycle. Temperature and air humidity were set to 22°C and 50% to 60%, respectively. The age of the mice ranked from 4 to 6 months at the start time of testing. The murine studies were based on power analyses to assure adequate sample sizes, performed in accordance with relevant guidelines and regulations, and approved by the Animal Ethics Committee of the Government of Upper Bavaria, Germany (animal protocol number VTA 55.2‐1‐54‐2532‐46‐16).

### 2.2 IntelliCage setup

Mice were group‐housed in an IntelliCage of TSE systems (IntelliCagePlus Version 3.3.2.0, TSE Systems) containing four testing corners, each equipped with two bottles, which allowed behavioral testing of the animals without any handling stress. Mice were identified via a subcutaneous implanted transponder and got assigned to one correct corner, where they could access via nose poke one of the two bottles. In the other three incorrect corners, access to the drinking bottles was denied. Observer‐independent behavior of the animals was tracked for 8 days by the provided computer software using subcutaneous transponders with ring antenna in the corners of the drinking bottles. The number of tongue‐contacts with spouts of the bottles was registered by a lickometer and displayed as number of licks. Chow food was provided ad libitum in the center of the IntelliCage.

#### 2.2.1 Two‐bottle sucrose versus water choice test

To adapt mice to a standard 10% sucrose solution used for the sucrose wanting test as hedonic incentive stimulus, animals got access for 5 s to sucrose solution in the respective correct corner after nose poke performance at both drinking bottles {Holter, 2015 #49}. For a habituation period of 24 hr, no plain water was available but sucrose solution. After this forced sucrose period, the test program was switched to a two‐bottle sucrose versus water choice test in the designated correct corner. Mice had access for 5 s to water or sucrose in the correct corner at the left or right side, respectively, after one single nose poke. During those 5 s, the doors to the bottles stayed open and allowed free interaction of the mice with the spout. A LED light indicated the sucrose containing side after entry of the correct corner. After each trial, mice had to leave the test corner before starting a new nose poke. Visits in correct and incorrect corners, first choice nose pokes, total numbers of nose pokes and licks for fluid in the respective sides were monitored for 5 days and analyzed. Nose pokes and visits are parameters for activity, licks for fluid are referred to as fluid consumption. Interactions are displayed in 2 hr intervals.

#### 2.2.2 Progressive ratio setup

The motivation of mice to consume sucrose rather than neutral water was measured in a two‐bottle sucrose versus water choice test under a progressive ratio (PR)‐2 paradigm for sucrose access. Animals had to perform increasing numbers of nose pokes in the respective correct corner in order to get access to the reinforcer sucrose solution for 5 s. Following every 10th correct reinforcer delivery, the number of nosepokes in the respective sucrose corner to earn the next reinforcer was increased by two. For accessing water, the fixed ratio of 1 nose poke was kept throughout the entire testing time. After each trial, mice had to leave the test corner before starting a new nose poke. The motivation for getting access to the reinforcer sucrose was measured with the progressive ratio scale for a period of eight nights.

### 2.3 Immunofluorescence

Mice were euthanized in CO_2_ and perfused through the heart using a peristaltic pump. After a washing step with ice cold PBS, mice were perfused with 4% paraformaldehyde (PFA) and the brains extracted and postfixed overnight before transferring them to 30% sucrose followed by PBS. Brains were cut coronally into 20 μm sections and treated with citrate‐containing heat‐induced epitope retrieval buffer for 30 min at 85°C. Sections were washed 3 × 5 min in PBS‐Triton and blocked in PBS‐Triton containing 1% FBS and 2% nonfat milk powder. Immunofluorescence staining against dopamine transporter (DAT) was conducted by an overnight incubation with anti‐DAT (1:200, Cat. #MAB369, Merck Millipore) primary antibody at 4°C, sections were subsequently washed 3 × 10 min in TBS. Alexa Fluor 568 goat antirat IgG (H + L) secondary antibody (Cat. A‐11077, Invitrogen Thermo Scientific) was used in the dilution 1:1,000 in blocking buffer and incubated for 1 hr at room temperature. Sections were washed again 3 × 10 min in TBS, mounted on glass slides, and dried at room temperature. Sections were covered in Elvanol mounting medium and sealed under a coverslip. Until imaging slides were stored in the dark at 4°C.

### 2.4 High caloric food ranking in humans

As previously reported (Kullmann et al., 2015), pictures of calorie‐dense, processed savory and sweet foods such as hamburger and donuts, respectively, were rated on a laptop on a scale of 1 to 9 (duration approx. 10 min). Ratings were performed under anorexigenic conditions (60 min after a nasal insulin bolus application). 63 participants rated 100 food pictures in two separate blocks according to explicit “liking” (“How much do you like the food item in general?”) and “wanting” (“How much would you like to eat the food item right now?”). For genotyping, DNA was isolated from whole blood using a commercial DNA isolation kit (NucleoSpin, Macherey & Nagel, Düren, Germany). The SNP rs2334499 was genotyped using the Agena MassARRAY^®^ System with iPLEX software (Agena Bioscience GmbH). All subjects provided informed written consent, and the local ethics committee at the University of Tübingen approved the protocol. Human studies were performed in accordance with the relevant local guidelines and regulations.

### 2.5 Statistical analysis

For statistical analyses, GraphPad Prism 8.0.1 was used. Multiple comparisons were performed by two‐way ANOVA with post hoc Sidak's multiple comparison tests. Two‐tailed unpaired Student's *t* tests were used to compare two groups. *p*‐values ≤ .05 were considered as statistically significant. All results are presented as means ± *SEM*.

## 3 RESULTS

### 3.1 Dusp8 KO mice have increased sucrose consumption in a self‐administration setup

**FIGURE 1:**
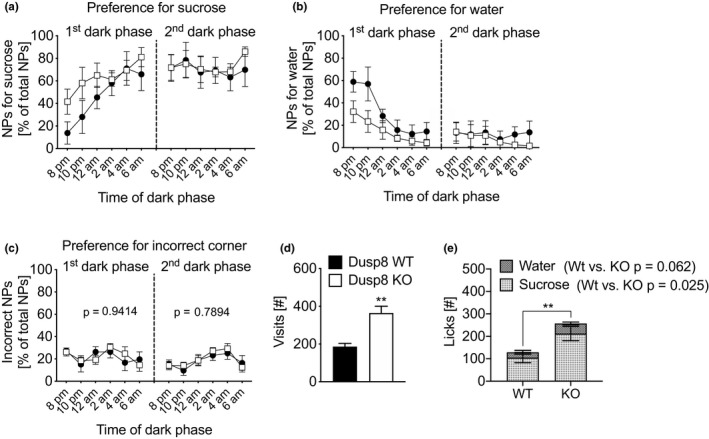
Two‐bottle sucrose versus. water choice test of female, chow‐fed Dusp8 WT & KO mice. Preference for (a) sucrose or (b) water in the first and second dark phase, displayed as percentage of nose pokes (NPs) for sucrose or water versus total NPs, respectively. (c) Preference for NPs in incorrect corners in the first and second dark phase. (d) Total number of corner visits in the second dark phase. (e) Total consumption of water and sucrose in the second dark phase. Female WT: *n* = 6, female Dusp8 KO: *n* = 8. Means ±*SEM*. **p < .01

To evaluate whether Dusp8 plays a role in sucrose reward and wanting, we conducted a self‐administered two‐bottle sucrose versus water choice test in the IntelliCage setup in chow‐fed female WT and Dusp8 KO mice littermates (WT: 21.7 ± 1.9 g BW; KO 21.4 ± 2.1 g BW). The preference for sucrose, measured as the percentage of nose pokes for sucrose of all performed nose pokes, was comparable in Dusp8 KO and WT mice. Both genotypes showed a comparable increased preference for sucrose in the first dark phase of the test paradigm (Figure 1a, ANOVA *p* = .22) that was consistent with a decreased preference for water (Figure 1b, ANOVA *p* = .66). The preference levels for both sucrose and water remained stable during the second (Figure 1a, ANOVA *p* = .81; Figure 1b, ANOVA *p* = .65) and the subsequent dark phases (data not shown). The preferences for nose pokes in incorrect corners did not differ between both genotypes and remained stable throughout the testing time (Figure 1c, first dark phase ANOVA *p* = .94, second dark phase ANOVA *p* = .78).

Similar to earlier observations by our laboratory (Baumann et al., 2019), we saw an overall higher locomotor activity of Dusp8 KO mice compared to Dusp8 WT controls that was reflected by a higher number of corner visits (Figure 1d). We further found an overall higher number of licks at the drinking bottles for Dusp8 KO mice. This increase was caused by a significantly higher number of nose pokes for sucrose (Figure 1e). The amount of consumed water did not differ between genotypes. Overall, our assessment of self‐administered sucrose consumption revealed comparable preferences for sucrose or water in the Dusp8 KO and WT mice. Nonetheless, females deficient for Dusp8 showed higher locomotor activity and an increased consumption of sucrose.

### 3.2 Dusp8 deficiency increases sucrose foraging but not the reinforcement value of sucrose in a progressive ratio operant licking test

The increased sucrose consumption of female Dusp8 KO mice prompted us to test their motivational behavior for sucrose in a progressive ratio reinforcement schedule. Specifically, to quantify the motivation of Dusp8 WT and KO mice for consuming sucrose, the mice had to increase their nose pokes in the correct corners after a correct trial to get access to the drinking bottles with sweet sucrose solution. The water bottles remained accessible with performing only one nose poke. With this approach, we measured the reinforcement value of sucrose, that is, how much effort the mice are willing to invest for consuming sucrose before switching to the easily accessible water.

**FIGURE 2:**
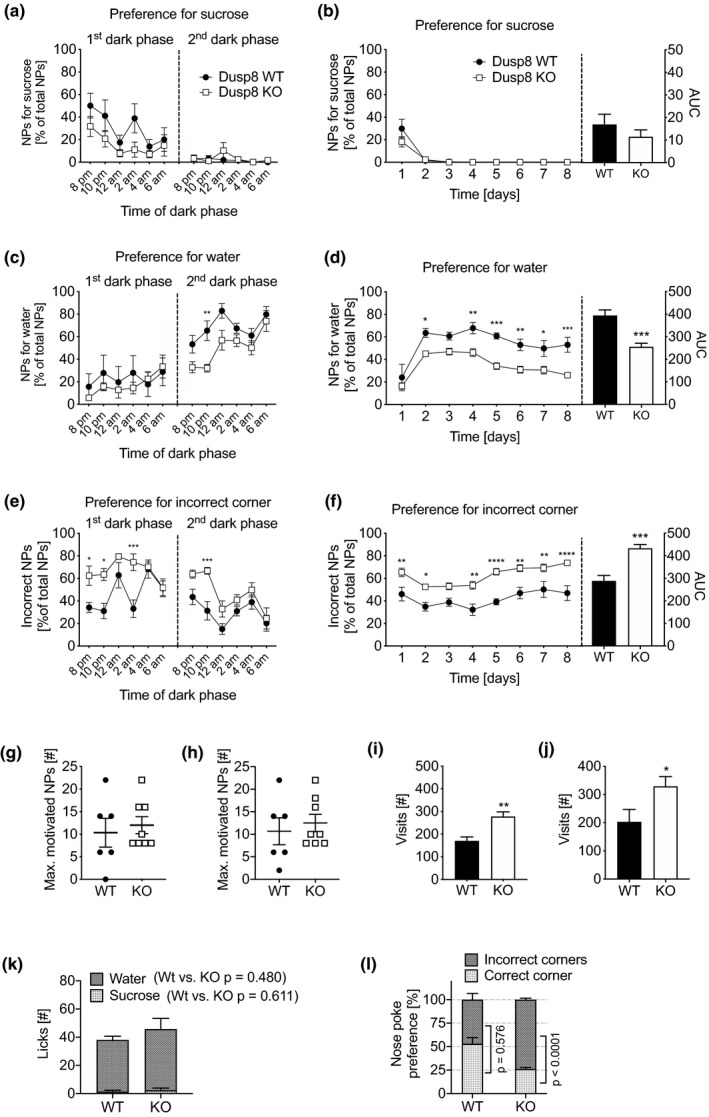
Sucrose preference was assessed in female chow‐fed Dusp8 WT and KO mice by a two‐bottle choice test for sucrose versus water with a progressive ratio schedule. Preference for nose poke (NP) performance for accessing sucrose in the (a) first and second night phase and (b) during the 8 days of testing, measured by the number of nose pokes (NPs) in the correct corner. Preference for NPs for water in the (c) first and second night phase and (d) during the 8 days of testing. Preference for NPs in the incorrect corner of the IntelliCage setup during the (e) first and second night phase and (f) over the 8 days of testing. Maximum numbers of NPs in the progressive ratio schedule (g) after the second night and (h) during the 8th dark phase of testing indicate a comparable motivation for sucrose consumption in WT and Dusp8 KO mice. Increased foraging and random investigation behaviors in Dusp8 KO mice were reflected by higher total numbers of corner visits in the second night (i) and during the 8th dark phase (j). Total liquid consumption in the second night phase remained unchanged (k). Dusp8 KO mice have a random 25% distribution in the nose poke preference for the assigned correct versus. incorrect corners during the 8th dark phase (l). Female WT: *n* = 6, female Dusp8 KO: *n* = 8. Means ± *SEM*. *p < .05, **p < .01, ***p < .001

After starting the motivation paradigm, within the first night the preference for sucrose dropped dramatically in both Dusp8 KO and WT mice, and remained at minimum levels throughout the entire testing phase of 8 days (Figure 2a,b). This finding showed that the reinforcement value of sucrose was not affected by Dusp8 ablation. However, from day 2 onwards Dusp8 WT mice showed a higher preference for water (Figure 2c,d), while Dusp8 KO mice had an elevated preference for incorrect nose pokes that was detached from the access to water or sucrose (Figure 2e,f). A higher preference for incorrect nose pokes may indicate elevated foraging, or motivation behavior in Dusp8 KO mice. However, the breakpoint as maximum number of motivated nose pokes for sucrose access was comparable in both genotypes at day 2 (Figure 2g) or day 8 (Figure 2h) of the testing paradigm. Increased numbers of overall corner visits of Dusp8 KO mice at day 2 (Figure 2i) and throughout the test time (Figure 2j) rather pointed toward elevated sucrose foraging behavior in Dusp8 KO females. On day 2, the amounts of consumed liquids were comparable between both genotypes (Figure 2k). Notably, while the percentage of entrained nose pokes in the correct test corner remained elevated in Dusp8 WT mice (Day 8, Figure 2l), we found a randomly distributed pattern of nose pokes, that is, 25% versus 75%, for the correct and the three incorrect corners in Dusp8 KO mice (Dusp8 WT: correct 53.0%, incorrect 47.0%, ±6.6%; Dusp8 KO: correct 26.2%, incorrect 73.8%, ±1.6%). Overall, we found a comparable reinforcement value of sucrose in Dusp8 KO and WT mice, but an elevated and, supposedly random, trial‐and‐error‐like foraging behavior in Dusp8 KO mice.

### 3.3 Incentive seeking strategy is independent of dopaminergic system in Dusp8 KO mice

**FIGURE 3:**
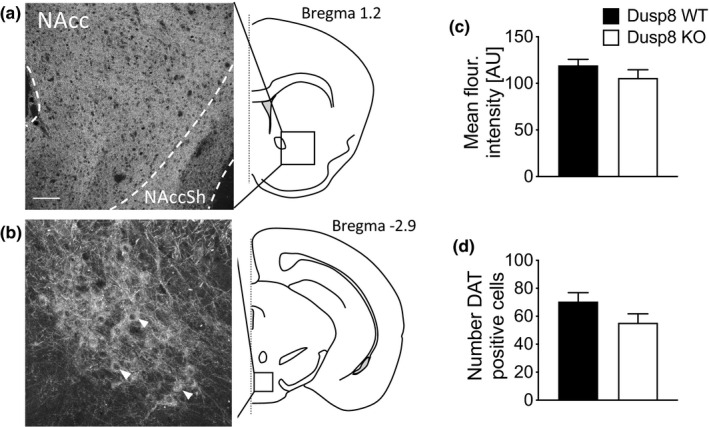
Dopamine transporter (DAT) densities in the NAcc (a) and the VTA (b), two key areas for sucrose reward, were assessed by immunostaining for DAT in female mice. (c) Signal intensities of DAT‐positive projections in the NAcc and (d) the number of DAT‐positive cells in the VTA of WT and Dusp8 KO females. Arrow heads indicate examples for DAT‐positive cells. NAcc: nucleus accumbens, NAccSh: nucleus accumbens shell. Female WT: *n* = 4, female Dusp8 KO: *n* = 6. Scale bar 100 µm. Means ± *SEM*

The dopaminergic reward system in the brain is crucially involved in motivation‐related and drive behavior (Berridge, 2007; Ferrario et al., 2016). The close network of the NAcc and the ventral tegmental area is regulating the onset of eating behavior and affects the salience of different nutritional stimuli introduced to the animal via dopamine (Nieh et al., 2016). Prompted by the different sucrose‐seeking strategy, we wanted to investigate the neuronal background of that behavior. We therefore analyzed the integrity of the dopaminergic reward system of the NAcc and the VTA and performed immunofluorescence staining against the dopamine transporter (Figure 3a,b). Therefore, we analyzed the integrity of dopaminergic projections to the NAcc and their cellular origins in the VTA. Overall, we found a comparable staining intensity in the NAcc (Figure 3c) and similar numbers of DAT‐positive cells in the ventral tegmental area (Figure 3d). This points toward an intact reward system in the striatum and an unaltered development and maturation process, respectively.

### 3.4 Association between *DUSP8* SNP rs2334499 and hedonic rating of sweet high caloric food

Mice with a deficiency in Dusp8 showed a higher consumption of sucrose, increased locomotion, and higher sucrose foraging behavior. Recently, we already reported increased locomotion, and further revealed higher anxiety levels and impaired spatial cognition in mice deficient for Dusp8 (Baumann et al., 2019). Consistent with our finding of decreased hippocampus mass and volume in Dusp8 KO mice, we moreover revealed that human carriers of *DUSP8* variant SNP rs2334499 have a lower volume of the hippocampus subregions subiculum and CA4 (Baumann et al., 2019). Prompted by this translational earlier study and our new finding of perturbed sucrose reward behavior in Dusp8 KO mice, we next aimed to assess whether human carriers of the rs2334499 diabetes‐risk allele show alterations in their preference for sweet high caloric food compared to human carriers of the major allele.

**FIGURE 4:**
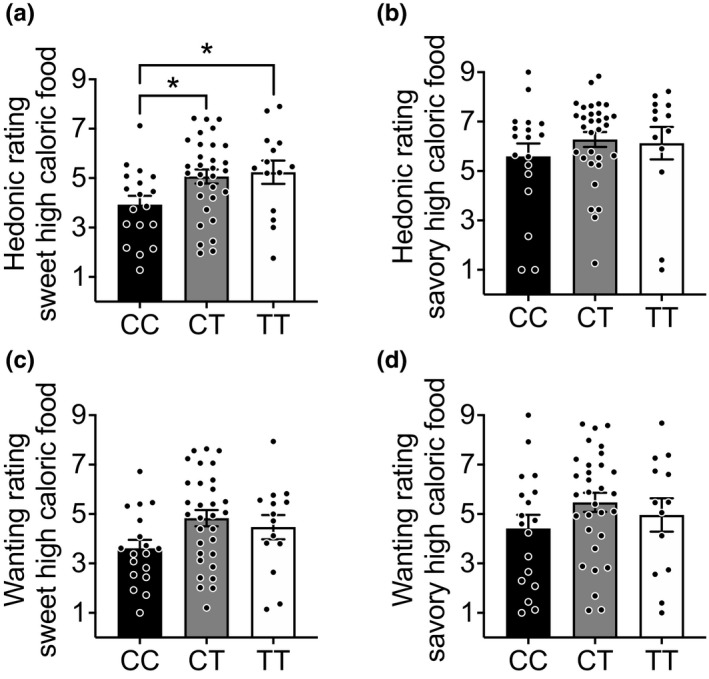
Rating for sweet and savory high caloric food in humans. Hedonic (liking) rating scores of carriers of the major C and minor T allele of *DUSP8* SNP rs2334499 of (a) sweet high caloric food (CC: *n* = 18, CT: *n* = 31, TT: *n* = 14) and (b) savory high caloric food (CC: *n* = 18, CT: *n* = 31, TT: *n* = 13). (c,d) show the wanting rating score for sweet and savory high caloric food, respectively. Means ± *SEM*. * *p* < .05

Specifically, we assessed the evaluation of visual food cues in sixty‐three participants by collecting a rating score for sweet high caloric versus savory high caloric food under standardized conditions. Minor T‐risk allele carriers rated sweet high caloric food higher compared to major allele CC carriers (Figure 4a). Hedonic rating scores for savory high caloric food did not differ between *DUSP8* genotypes (Figure 4b). Moreover, the wanting levels for sweet high caloric food (Figure 4c) or savory high caloric food (Figure 4d) were comparable between genotypes. Taken together, human carriers of the minor allele of the *DUSP8* SNP rs2334499, which has previously been linked to T2D risk (Kong et al., 2009; Morris et al., 2012), hypothalamic insulin resistance (Schriever et al., 2020) and to a smaller hippocampal subiculum and CA4 layer volume (Baumann et al., 2019), show a preference for sweet high caloric food compared to major allele carriers.

## 4 DISCUSSION

We here expand our earlier work that showed a role for the MAPK‐specific Dusp8 as gatekeeper for insulin resistance, cognition, and anxiety behavior in mice (Baumann et al., 2019; Schriever et al., 2020). We now reveal an additional role for Dusp8 in controlling sucrose reward behavior. Female chow‐fed Dusp8 KO mice showed unaltered sucrose preference in a progressive ratio setup, but perturbed sucrose‐seeking behavior. These murine data are further consistent with our new translational finding that human carriers of the *DUSP8* SNP rs2334499 minor allelic variant have higher hedonic ratings for sweet high caloric food compared to major allele carriers.

In female mice with Dusp8 deficiency, we found unperturbed sucrose preference but a change in their sucrose‐seeking strategy. Most interestingly, we found a random distribution pattern of foraging to correct and incorrect corners in our Dusp8 KO mice subjected to a progressive ratio task, while total water intake was unchanged. This “trial and error” strategy to find sucrose in any corner was in stark contrast to WT control mice that still preferred the correct corner. This different strategy may indicate a negatively reinforcing, stereotypical behavior, and displacement activity driven by Dusp8 ablation. The brain region responsible for this increased displacement activity in Dusp8 KO mice remains unclear. Dopaminergic neurons in the reward‐related regions NAcc and VTA are potential candidates as they play a predominant role in regulating hedonia and sucrose reward behavior (Nieh et al., 2015). For instance, dopamine transporter (DAT)‐altered mice were shown to have higher motivational levels for sucrose consumption in a progressive ratio task (Davis et al., 2018). However, using immunohistochemical stainings in the NAcc and VTA, we found comparable DAT levels in Dusp8 WT and KO mice. Alterations in the reward‐seeking behavior observed in Dusp8 KO mice are thus unlikely to involve morphological differences in dopamine circuits in the VTA/NAcc or its efferent or afferent projections.

Recently, we reported that Dusp8 KO mice have a reduced hippocampal mass and volume and a mildly impaired spatial memory when challenged with a spatial learning paradigm (Baumann et al., 2019). In rodents, spatial and working memory processes are directly modulated by the availability, or absence, of sucrose (Kendig, 2014). A hippocampus‐striatal axis was further associated with reward behaviors (Nauta & Domesick, 1984) and learning, prediction and goal‐directed behaviors (Pennartz et al., 2011). Accordingly, a role of Dusp8 in this hippocampal‐striatal control of sucrose‐seeking behavior seems possible. Future studies should clarify whether sucrose restriction in the progressive ratio paradigm can exacerbate baseline spatial orientation deficits present in Dusp8 deficient mice. Such studies should further address whether hypercorticosteronemia, which we recently reported for male Dusp8 KO mice (Schriever et al., 2020), is a unifying determinant for the lower hippocampal mass, for the impairment in spatial orientiation, and for the aberrant sucrose‐seeking behavior.

Additional limitations of our studies are based on the choice of behavior tests, and on the paucity of evidence in respect to molecular mechanisms. Using the IntelliCage system with its group housing restricted our behavioral studies to female mice due to animal welfare guidelines and the higher aggression of males. Moreover, we did not control for the estrous cycle of our female mice, which may affect sucrose reward behaviors (Becker & Koob, 2016). Our IntelliCage setup, with its constant social interactions, avoids social deprivation and allows the study of complex murine behaviors in an unrestrained, home‐cage environment. It nonetheless adds a layer of complexity that may impede the interpretability of the results. Traditional testing of individual mice in Operant conditioning boxes could circumvent that issue and further allow testing of male mice. It would be interesting to compare results collected in IntelliCages versus Operant conditioning boxes, to learn whether pre‐existing pathologies such as the hypercorticosteronemia, the impaired spatial orientation, or the anxiety (Baumann et al., 2019; Schriever et al., 2020) of Dusp8 KO mice can differentially affect sucrose reward behaviors under single‐housed versus group‐housed conditions. The study design could further be optimized to test the reinforcer efficacy of sucrose in more detail by using varying doses of sucrose. Similarly, Dusp8 WT and KO mice could be tested for differences in sweet perception (Dotson & Spector, 2007), or for parameters classically used to distinguish the hedonic categories wanting and liking, that is, facial expression or licking patterns of lips and paws (Berridge, 2000). Last, future studies should aim to delineate the involvement of MAP kinases and in general the molecular mechanisms that drive the sucrose‐seeking behavior of Dusp8 KO mice.

In Dusp8 KO mice, we could not discriminate whether the increased consumption of sucrose is driven by an incentive salience stimulus in the terminology of “wanting” and “liking”. However, by assessing the evaluation of visual food cues in sixty‐three participants via a rating score for sweet versus savory high caloric food, we could show that human carriers of *DUSP8* variant SNP rs2334499 have an increased liking for sweet high caloric food whereas wanting scores were not differing between *DUSP8* genotypes. Of note, an association of visual food cue reactivity and eating behavior has been reported before (Boswell & Kober, 2016), but the methodology used for assessing wanting versus liking is controversially discussed (Tibboel et al., 2015). By tightly keeping our participants in a comparable metabolic state to eliminate a bias in rating score by differences in alliesthesia levels, we were nonetheless able to strengthen the validity of our results. Overall, we report higher liking scores for sweet high caloric food in participants carrying the diabetes‐risk variant. Whether such an increased liking leads to an actual higher consumption of sweet high caloric food, potentially even to the increased risk for T2D (Kong et al., 2009; Morris et al., 2012), remains to be tested.

## 5 CONCLUSION

Taken together, we show an involvement of Dusp8 in regulating sucrose consumption and sucrose‐seeking behaviors in mice that appear to be independent from perturbations in mesolimbic dopaminergic circuitry. Our data are consistent with our earlier work (Baumann et al., 2019; Schriever et al., 2020) and suggest that this MAPK‐specific phosphatase is involved in a wide range of translationally relevant, stimuli‐triggered behaviors via yet unknown mechanisms. Variation in *DUSP8* further appears to be involved in mediating hedonic taste sensation without altering affect in humans. The increased preference for sweet high caloric food in Dusp8 minor allele adds to earlier work on the association of SNP rs2334499 minor allele with the overall type 2 diabetes risk (Kong et al., 2009; Morris et al., 2012) or the increased hypothalamic insulin resistance (Schriever et al., 2020). Collectively, these data suggest a translational role for this genetic locus in the development of diabetes type 2, likely driven by mechanisms orchestrated within the CNS.

## CONFLICT OF INTEREST

Dr. Matthias Tschöp is a member of the scientific advisory board of ERX Pharmaceuticals, Inc., Cambridge, MA. He was a member of the Research Cluster Advisory Panel (ReCAP) of the Novo Nordisk Foundation between 2017 and 2019. He attended a scientific advisory board meeting of the Novo Nordisk Foundation Center for Basic Metabolic Research, University of Copenhagen, in 2016. He received funding for his research projects by Novo Nordisk (2016–2020) and Sanofi‐Aventis (2012–2019). He was a consultant for Bionorica SE (2013–2017), Menarini Ricerche S.p.A. (2016), and Bayer Pharma AG Berlin (2016). As former Director of the Helmholtz Diabetes Center and the Institute for Diabetes and Obesity at Helmholtz Zentrum München (2011–2018) and since 2018 as CEO of Helmholtz Zentrum München he has been responsible for collaborations with a multitude of companies and institutions, worldwide. In this capacity, he discussed potential projects with and has signed/signs contracts for his institute(s) and for the staff for research funding and/or collaborations with industry and academia, worldwide, including but not limited to pharmaceutical corporations like Boehringer Ingelheim, Eli Lilly, Novo Nordisk, Medigene, Arbormed, BioSyngen, and others. In this role, he was/is further responsible for commercial technology transfer activities of his institute(s), including diabetes‐related patent portfolios of Helmholtz Zentrum München, for example, WO/2016/188932 A2 or WO/2017/194499 A1. Dr. Tschöp confirms that to the best of his knowledge none of the above funding sources were involved in the preparation of this paper.

## AUTHOR CONTRIBUTIONS

PB and SCS performed murine experiments and collected tissues. IntelliCage experiments were performed by PB. PB conducted cell counting and image analysis. PB, AZ, HF, VGD, MHdA, WW, and SMH designed and planned the behavioral experiments. PB, AZ, and SMH analyzed the sucrose preference and progressive ratio experiments. SK, AP, and MH performed the analysis of the human data set. MHT was involved in the framework and supervision of the project. PB, SCS, and PTP developed the conceptual framework of the study. PB, SCS, MH, SK, and PTP prepared the manuscript. This work was supported in part by the Helmholtz Portfolio Program “Metabolic Dysfunction” (MHT), by ExNet‐0041‐Phase2‐3 (SyNergy‐HMGU, WW), by the AMPro project—“Aging and Metabolic Programming” (WW), by the Alexander von Humboldt Foundation (MHT), by the Helmholtz Alliance ICEMED‐Imaging and Curing Environmental Metabolic Diseases (WW, SCS, MHT), by the German Center for Diabetes Research (DZD; SCS, PP, MH, MHA), by the Helmholtz‐Israel‐Cooperation in Personalized Medicine (PP), by the Helmholtz Initiative for Personalized Medicine (iMed; MHT), by the German Federal Ministry of Education and Research “01KX1012” (MHA) and through the Initiative and Networking Fund of the Helmholtz Association (WW; MHT).

### Peer Review

The peer review history for this article is available at https://publons.com/publon/10.1002/brb3.1928.

## Data Availability

The data that support the findings of this study are available from the corresponding author upon reasonable request.

## References

[brb31928-bib-0001] Baumann, P. , Schriever, S. C. , Kullmann, S. , Zimprich, A. , Feuchtinger, A. , Amarie, O. , Peter, A. , Walch, A. , Gailus‐Durner, V. , Fuchs, H. , Hrabě de Angelis, M. , Wurst, W. , Tschöp, M. H. , Heni, M. , Hölter, S. M. , & Pfluger, P. T. (2019). Dusp8 affects hippocampal size and behavior in mice and humans. Scientific Reports, 9(1), 19483 10.1038/s41598-019-55527-7 31862894 PMC6925303

[brb31928-bib-0002] Becker, J. B. , & Koob, G. F. (2016). Sex differences in animal models: Focus on addiction. Pharmacological Reviews, 68(2), 242–263. 10.1124/pr.115.011163 26772794 PMC4813426

[brb31928-bib-0003] Berridge, K. C. (2000). Measuring hedonic impact in animals and infants: Microstructure of affective taste reactivity patterns. Neuroscience and Biobehavioral Reviews, 24(2), 173–198. 10.1016/S0149-7634(99)00072-X 10714382

[brb31928-bib-0004] Berridge, K. C. (2007). The debate over dopamine's role in reward: The case for incentive salience. Psychopharmacology (Berl), 191(3), 391–431. 10.1007/s00213-006-0578-x 17072591

[brb31928-bib-0005] Berridge, K. C. , Ho, C. Y. , Richard, J. M. , & DiFeliceantonio, A. G. (2010). The tempted brain eats: Pleasure and desire circuits in obesity and eating disorders. Brain Research, 1350, 43–64. 10.1016/j.brainres.2010.04.003 20388498 PMC2913163

[brb31928-bib-0006] Berthoud, H. R. , Lenard, N. R. , & Shin, A. C. (2011). Food reward, hyperphagia, and obesity. American Journal of Physiology: Regulatory, Integrative and Comparative Physiology, 300(6), R1266–R1277. 10.1152/ajpregu.00028.2011 PMC3119156 21411768

[brb31928-bib-0007] Boswell, R. G. , & Kober, H. (2016). Food cue reactivity and craving predict eating and weight gain: A meta‐analytic review. Obesity Reviews, 17(2), 159–177. 10.1111/obr.12354 26644270 PMC6042864

[brb31928-bib-0008] Castro, D. C. , & Berridge, K. C. (2014). Advances in the neurobiological bases for food 'liking' versus 'wanting'. Physiology & Behavior, 136, 22–30. 10.1016/j.physbeh.2014.05.022 24874776 PMC4246030

[brb31928-bib-0009] Davis, G. L. , Stewart, A. , Stanwood, G. D. , Gowrishankar, R. , Hahn, M. K. , & Blakely, R. D. (2018). Functional coding variation in the presynaptic dopamine transporter associated with neuropsychiatric disorders drives enhanced motivation and context‐dependent impulsivity in mice. Behavioral Brain Research, 337, 61–69. 10.1016/j.bbr.2017.09.043 PMC5645257 28964912

[brb31928-bib-0010] Dotson, C. D. , & Spector, A. C. (2007). Behavioral discrimination between sucrose and other natural sweeteners in mice: Implications for the neural coding of T1R ligands. Journal of Neuroscience, 27(42), 11242–11253. 10.1523/JNEUROSCI.1227-07.2007 17942718 PMC6673039

[brb31928-bib-0011] Fagherazzi, G. , Vilier, A. , Saes Sartorelli, D. , Lajous, M. , Balkau, B. , & Clavel‐Chapelon, F. (2013). Consumption of artificially and sugar‐sweetened beverages and incident type 2 diabetes in the Etude Epidemiologique aupres des femmes de la Mutuelle Generale de l'Education Nationale‐European Prospective Investigation into Cancer and Nutrition cohort. American Journal of Clinical Nutrition, 97(3), 517–523. 10.3945/ajcn.112.050997 23364017

[brb31928-bib-0012] Farooq, A. , & Zhou, M. M. (2004). Structure and regulation of MAPK phosphatases. Cellular Signalling, 16(7), 769–779. 10.1016/j.cellsig.2003.12.008 15115656

[brb31928-bib-0013] Ferrario, C. R. , Labouebe, G. , Liu, S. , Nieh, E. H. , Routh, V. H. , Xu, S. , & O'Connor, E. C. (2016). Homeostasis Meets Motivation in the Battle to Control Food Intake. Journal of Neuroscience, 36(45), 11469–11481. 10.1523/jneurosci.2338-16.2016 27911750 PMC5125214

[brb31928-bib-0014] Imamura, F. , O'Connor, L. , Ye, Z. , Mursu, J. , Hayashino, Y. , Bhupathiraju, S. N. , & Forouhi, N. G. (2016). Consumption of sugar sweetened beverages, artificially sweetened beverages, and fruit juice and incidence of type 2 diabetes: Systematic review, meta‐analysis, and estimation of population attributable fraction. British Journal of Sports Medicine, 50(8), 496–504. 10.1136/bjsports-2016-h3576rep 27044603 PMC4853528

[brb31928-bib-0015] Kanoski, S. E. , & Grill, H. J. (2017). Hippocampus contributions to food intake control: Mnemonic, neuroanatomical, and endocrine mechanisms. Biological Psychiatry, 81(9), 748–756. 10.1016/j.biopsych.2015.09.011 26555354 PMC4809793

[brb31928-bib-0016] Kendig, M. D. (2014). Cognitive and behavioural effects of sugar consumption in rodents. A review. Appetite, 80, 41–54. 10.1016/j.appet.2014.04.028 24816323

[brb31928-bib-0017] Kong, A. , Steinthorsdottir, V. , Masson, G. , Thorleifsson, G. , Sulem, P. , Besenbacher, S. , Jonasdottir, A. , Sigurdsson, A. , Kristinsson, K. T. , Jonasdottir, A. , Frigge, M. L. , Gylfason, A. , Olason, P. I. , Gudjonsson, S. A. , Sverrisson, S. , Stacey, S. N. , Sigurgeirsson, B. , Benediktsdottir, K. R. , Sigurdsson, H. , … Stefansson, K. (2009). Parental origin of sequence variants associated with complex diseases. Nature, 462(7275), 868–874. 10.1038/nature08625 20016592 PMC3746295

[brb31928-bib-0018] Krentz, N. A. J. , & Gloyn, A. L. (2020). Insights into pancreatic islet cell dysfunction from type 2 diabetes mellitus genetics. Nature Reviews Endocrinology, 16(4), 202–212. 10.1038/s41574-020-0325-0 32099086

[brb31928-bib-0019] Kullmann, S. , Heni, M. , Veit, R. , Scheffler, K. , Machann, J. , Häring, H.‐U. , Fritsche, A. , & Preissl, H. (2015). Selective insulin resistance in homeostatic and cognitive control brain areas in overweight and obese adults. Diabetes Care, 38(6), 1044–1050. 10.2337/dc14-2319 25795413

[brb31928-bib-0020] Kullmann, S. , Kleinridders, A. , Small, D. M. , Fritsche, A. , Haring, H. U. , Preissl, H. , & Heni, M. (2020). Central nervous pathways of insulin action in the control of metabolism and food intake. The Lancet Diabetes & Endocrinology, 8(6), 524–534. 10.1016/S2213-8587(20)30113-3 32445739

[brb31928-bib-0021] Liu, R. , van Berlo, J. H. , York, A. J. , Vagnozzi, R. J. , Maillet, M. , & Molkentin, J. D. (2016). DUSP8 regulates cardiac ventricular remodeling by altering ERK1/2 signaling. Circulation Research, 119(2), 249–260. 10.1161/circresaha.115.308238 27225478 PMC4938738

[brb31928-bib-0022] Martell, K. J. , Seasholtz, A. F. , Kwak, S. P. , Clemens, K. K. , & Dixon, J. E. (1995). hVH‐5: A protein tyrosine phosphatase abundant in brain that inactivates mitogen‐activated protein kinase. Journal of Neurochemistry, 65(4), 1823–1833. 10.1046/j.1471-4159.1995.65041823.x 7561881

[brb31928-bib-0023] Morris, A. P. , Voight, B. F. , Teslovich, T. M. , Ferreira, T. , Segre, A. V. , Steinthorsdottir, V. , Strawbridge, R. J. , Khan, H. , Grallert, H. , Mahajan, J. , Prokopenko, I. , Kang, H. M. , Dina, C. , Esko, T. , Fraser, R. M. , Kanoni, S. , Kumar, A. , Lagou, V. , Langenburg, C. , … Diabetics Genetics Replication and Meta‐Analysis (DIAGRAM) Consortium (2012). Large‐scale association analysis provides insights into the genetic architecture and pathophysiology of type 2 diabetes. Nature Genetics, 44(9), 981–990. 10.1038/ng.2383 22885922 PMC3442244

[brb31928-bib-0024] Muda, M. , Theodosiou, A. , Rodrigues, N. , Boschert, U. , Camps, M. , Gillieron, C. , Davies, K. , Ashworth, A. , & Arkinstall, S. (1996). The dual specificity phosphatases M3/6 and MKP‐3 are highly selective for inactivation of distinct mitogen‐activated protein kinases. Journal of Biological Chemistry, 271(44), 27205–27208. 10.1074/jbc.271.44.27205 8910287

[brb31928-bib-0025] Nauta, W. J. , & Domesick, V. B. (1984). Afferent and efferent relationships of the basal ganglia. Ciba Foundation Symposium, 107, 3–29. 6437774 10.1002/9780470720882.ch2

[brb31928-bib-0026] Nieh, E. H. , Matthews, G. A. , Allsop, S. A. , Presbrey, K. N. , Leppla, C. A. , Wichmann, R. , Neve, R. , Wildes, C. P. , & Tye, K. M. (2015). Decoding neural circuits that control compulsive sucrose seeking. Cell, 160(3), 528–541. 10.1016/j.cell.2015.01.003 25635460 PMC4312417

[brb31928-bib-0027] Nieh, E. H. , Vander Weele, C. M. , Matthews, G. A. , Presbrey, K. N. , Wichmann, R. , Leppla, C. A. , Izadmehr, E. M. , & Tye, K. M. (2016). Inhibitory input from the lateral hypothalamus to the ventral tegmental area disinhibits dopamine neurons and promotes behavioral activation. Neuron, 90(6), 1286–1298. 10.1016/j.neuron.2016.04.035 27238864 PMC4961212

[brb31928-bib-0028] Pennartz, C. M. , Ito, R. , Verschure, P. F. , Battaglia, F. P. , & Robbins, T. W. (2011). The hippocampal‐striatal axis in learning, prediction and goal‐directed behavior. Trends in Neurosciences, 34(10), 548–559. 10.1016/j.tins.2011.08.001 21889806

[brb31928-bib-0029] Qi, Q. , Chu, A. Y. , Kang, J. H. , Jensen, M. K. , Curhan, G. C. , Pasquale, L. R. , Ridker, P. M. , Hunter, D. J. , Willett, W. C. , Rimm, E. B. , Chasman, D. I. , Hu, F. B. , & Qi, L. U. (2012). Sugar‐sweetened beverages and genetic risk of obesity. New England Journal of Medicine, 367(15), 1387–1396. 10.1056/NEJMoa1203039 PMC3518794 22998338

[brb31928-bib-0030] Robinson, T. E. , & Berridge, K. C. (1993). The neural basis of drug craving: An incentive‐sensitization theory of addiction. Brain Research. Brain Research Reviews, 18(3), 247–291. 10.1016/0165-0173(93)90013-P 8401595

[brb31928-bib-0031] Romaguera, D. , Norat, T. , Wark, P. A. , Vergnaud, A. C. , Schulze, M. B. , van Woudenbergh, G. J. , & Wareham, N. J. (2013). Consumption of sweet beverages and type 2 diabetes incidence in European adults: Results from EPIC‐InterAct. Diabetologia, 56(7), 1520–1530. 10.1007/s00125-013-2899-8 23620057

[brb31928-bib-0032] Ruud, J. , Steculorum, S. M. , & Bruning, J. C. (2017). Neuronal control of peripheral insulin sensitivity and glucose metabolism. Nature Communications, 8, 15259 10.1038/ncomms15259 PMC5418592 28469281

[brb31928-bib-0033] Schriever, S. C. , Kabra, D. G. , Pfuhlmann, K. , Baumann, P. , Baumgart, E. V. , Nagler, J. , Seebacher, F. , Harrison, L. , Irmler, M. , Kullmann, S. , Corrêa‐da‐Silva, F. , Giesert, F. , Jain, R. , Schug, H. , Castel, J. , Martinez, S. , Wu, M. , Häring, H.‐U. , de Angelis, M. H. , … Pfluger, P. T. (2020). Type 2 diabetes risk gene Dusp8 regulates hypothalamic Jnk signaling and insulin sensitivity. Journal of Clinical Investigation, 136363 10.1172/JCI136363 PMC7598066 32780722

[brb31928-bib-0034] Sescousse, G. , Caldu, X. , Segura, B. , & Dreher, J. C. (2013). Processing of primary and secondary rewards: A quantitative meta‐analysis and review of human functional neuroimaging studies. Neuroscience and Biobehavioral Reviews, 37(4), 681–696. 10.1016/j.neubiorev.2013.02.002 23415703

[brb31928-bib-0035] Stemmer, K. , Muller, T. D. , DiMarchi, R. D. , Pfluger, P. T. , & Tschop, M. H. (2019). CNS‐targeting pharmacological interventions for the metabolic syndrome. Journal of Clinical Investigation, 129(10), 4058–4071. 10.1172/JCI129195 PMC6763237 31380808

[brb31928-bib-0036] Thierry, A. M. , Gioanni, Y. , Degenetais, E. , & Glowinski, J. (2000). Hippocampo‐prefrontal cortex pathway: Anatomical and electrophysiological characteristics. Hippocampus, 10(4), 411–419. 10.1002/1098-1063(2000)10:4<411:aid-hipo7>3.0.co;2-a 10985280

[brb31928-bib-0037] Tibboel, H. , De Houwer, J. , & Van Bockstaele, B. (2015). Implicit measures of "wanting" and "liking" in humans. Neuroscience and Biobehavioral Reviews, 57, 350–364. 10.1016/j.neubiorev.2015.09.015 26432503

[brb31928-bib-0038] Tracy, A. L. , Jarrard, L. E. , & Davidson, T. L. (2001). The hippocampus and motivation revisited: Appetite and activity. Behavioral Brain Research, 127(1–2), 13–23. 10.1016/s0166-4328(01)00364-3 11718882

[brb31928-bib-0039] Turton, R. , Chami, R. , & Treasure, J. (2017). Emotional eating, binge eating and animal models of binge‐type eating disorders. Current Obesity Reports, 6(2), 217–228. 10.1007/s13679-017-0265-8 28434108

